# Supplementary material for: Structural basis of Ty3 retrotransposon integration at RNA Polymerase III-transcribed genes
Source: Nat Commun. 2021 Nov 30;12:6992. doi: 10.1038/s41467-021-27338-w (PMC8632968; doi:10.1038/s41467-021-27338-w)
Supplement: Supplementary file 1 — Supplementary Information [file 41467_2021_27338_MOESM1_ESM.pdf]

Supplementary Information

Structural basis of Ty3 retrotransposon integration at RNA Polymerase III-transcribed genes

Abascal-Palacios, et al.

Includes:

Supplementary Figures 1-6

Supplementary Table 1

Supplementary Figure 1. Reconstitution of the integration machinery and targeting complexes.

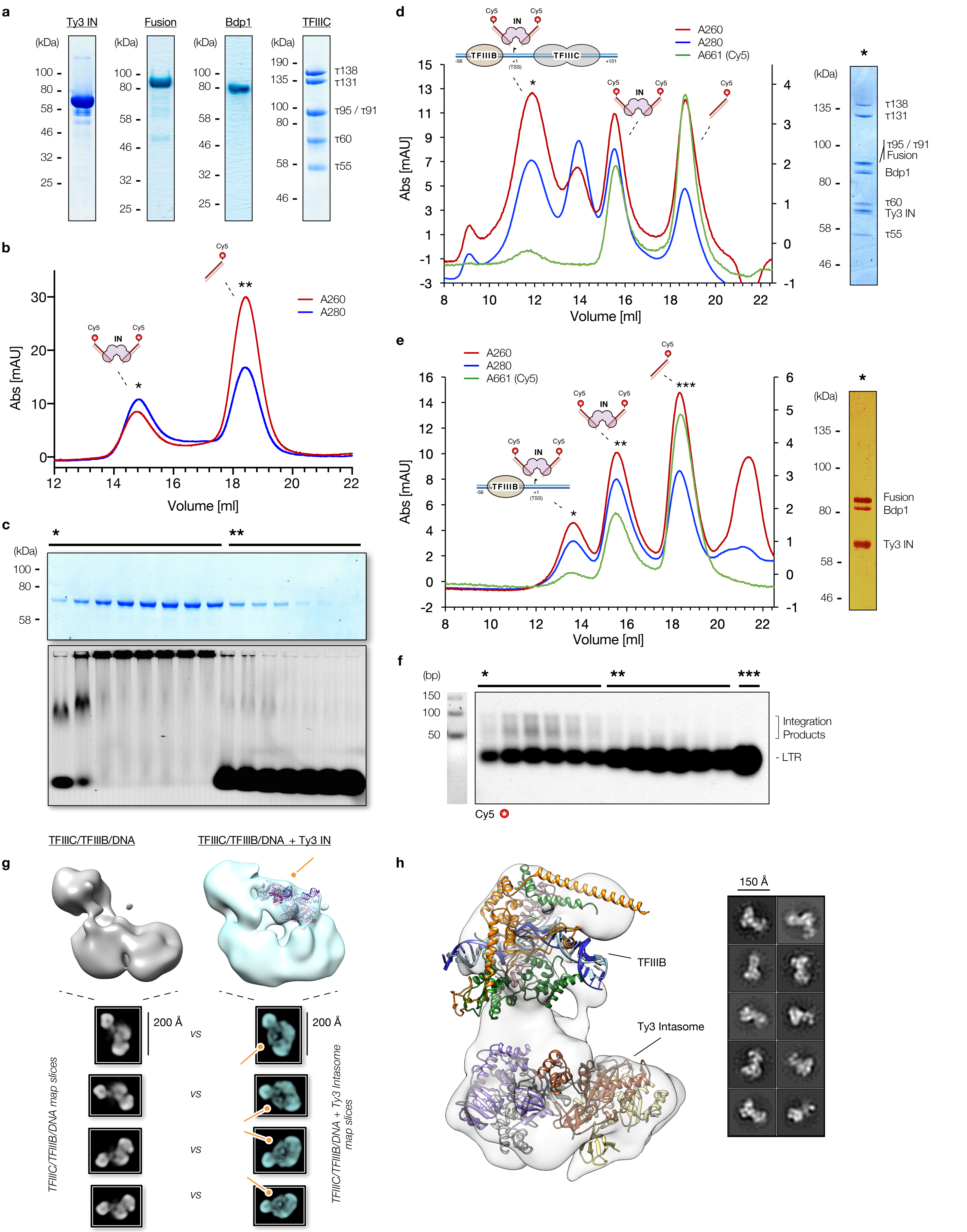

a, Purification of Ty3 integrase (Ty3 IN), Brf1-TBP fusion protein (Fusion), Bdp1 and TFIIIC components. Representative bands from SDS-PAGE analysis are shown. Each purification was repeated independently at least 5 times. **b,** Gel-filtration chromatography of Ty3 intasome. UV absorbance at 260 and 280 nm are indicated as red and blue lines, respectively. **c,** Analysis of the gel-filtration fractions by SDS-PAGE *(top)* showed the presence of Ty3 integrase only in the first elution peak. Analysis in 4.5% agarose gels *(bottom)* found free LTR in the second peak and the formation of a higher order complex in the first elution peak, suggesting the presence of Ty3 intasome in these fractions. Intasome reconstitution assays were repeated in triplicate. **d,** Gel-filtration chromatography of the full Ty3 integration machinery (TFIIIC/TFIIIB/DNA/Intasome). *Inset*, SDS-PAGE analysis of the first peak (*) confirmed the presence of all the components. Reconstitution assays were repeated in triplicate. **e,** Gel-filtration chromatography of the minimal Ty3 integration machinery (TFIIIB/DNA/Intasome). *Inset,* SDS-PAGE analysis of the first peak (*) confirmed the presence of all the components. Reconstitution assays were repeated in triplicate. **f,** Evaluation of the integration activity of the minimal Ty3 integration machinery. DNA products were analysed in 4.5% agarose gels after proteinase K treatment. Integration activity assays were repeated in triplicate. **g,** Negative stain electron microscopy analysis of TFIIIC/TFIIIB/DNA/Intasome complex. Comparison of EM reconstructions obtained in the absence *(left)* and presence *(right)* of Ty3 intasome shows the existence of an extra density on the periphery of the complex. Fitting of a homology model of Ty3 intasome (ribbon, 240 kDa) into the extra density shows a size compatible with a Ty3 integrase tetramer. Four independent central slices of the negative stain maps *(insets)* also confirm the presence of the extra region (orange arrows). **h,** Negative stain reconstruction of the minimal integration machinery. *S. cerevisiae* TFIIIB and a homology model of Ty3 intasome (4 subunits) are fitted in the map. Representative 2D class-averages (obtained from thousands of particles) are also shown *(inset)*.

Supplementary Figure 2. Cryo-EM data processing and resolution estimation

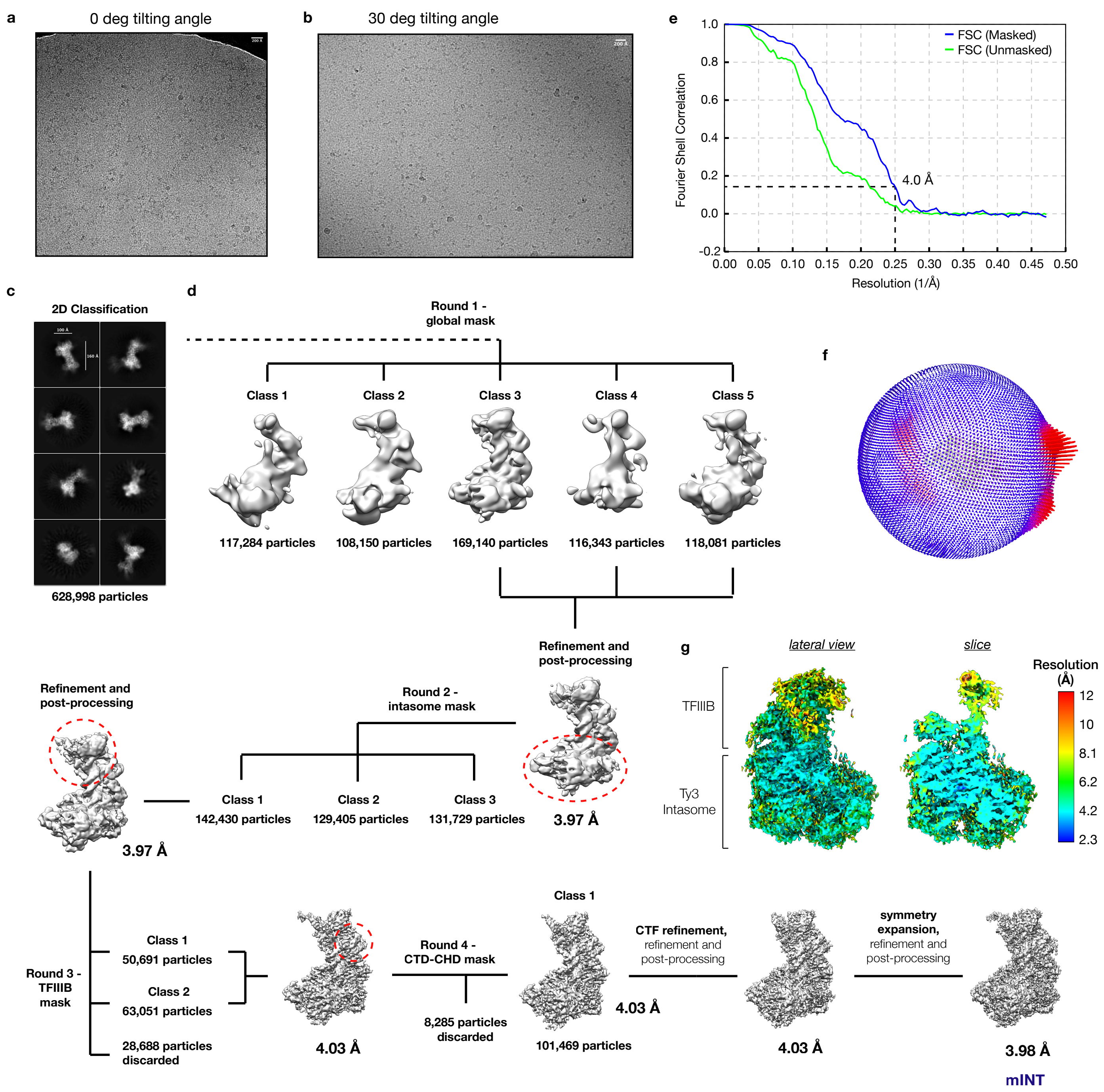

a, b, Representative raw micrographs of TFIIIB - Ty3 Intasome datasets collected at 0° tilting angle (**a**, 4974 micrographs) and 30° tilting angle (**b**, 2437 micrographs). **c,** Eight reference-free 2D class averages obtained from 628,998 independent particles. **d,** 3D classification of the joined set of particles from 0° and 30° tilting angles datasets. The particles were subjected to a hierarchical process, encompassing several rounds of classification using global or focused masks (dashed red circles around specific regions of the complex), as described in the schematic. The estimated resolution at the gold-standard FSC (FSC= 0.143) and the number of particles contributing to each class are indicated close to the corresponding 3D reconstructions. **e,** Fourier-shell correlation (FSC) representation of the masked (blue) and unmasked (green) cryo-EM reconstruction with the estimated resolution at the gold-standard FSC. **f,** Orientation distribution sphere of the particles that contributed to the final cryo-EM reconstruction. **g,** Resolution estimation of the cryo-EM map calculated with ResMap. Lateral *(left)* and central slice *(right)* views are shown and coloured according to the local resolution, as indicated in the scale bar.

Supplementary Figure 3. Cryo-EM reconstruction and model fitting

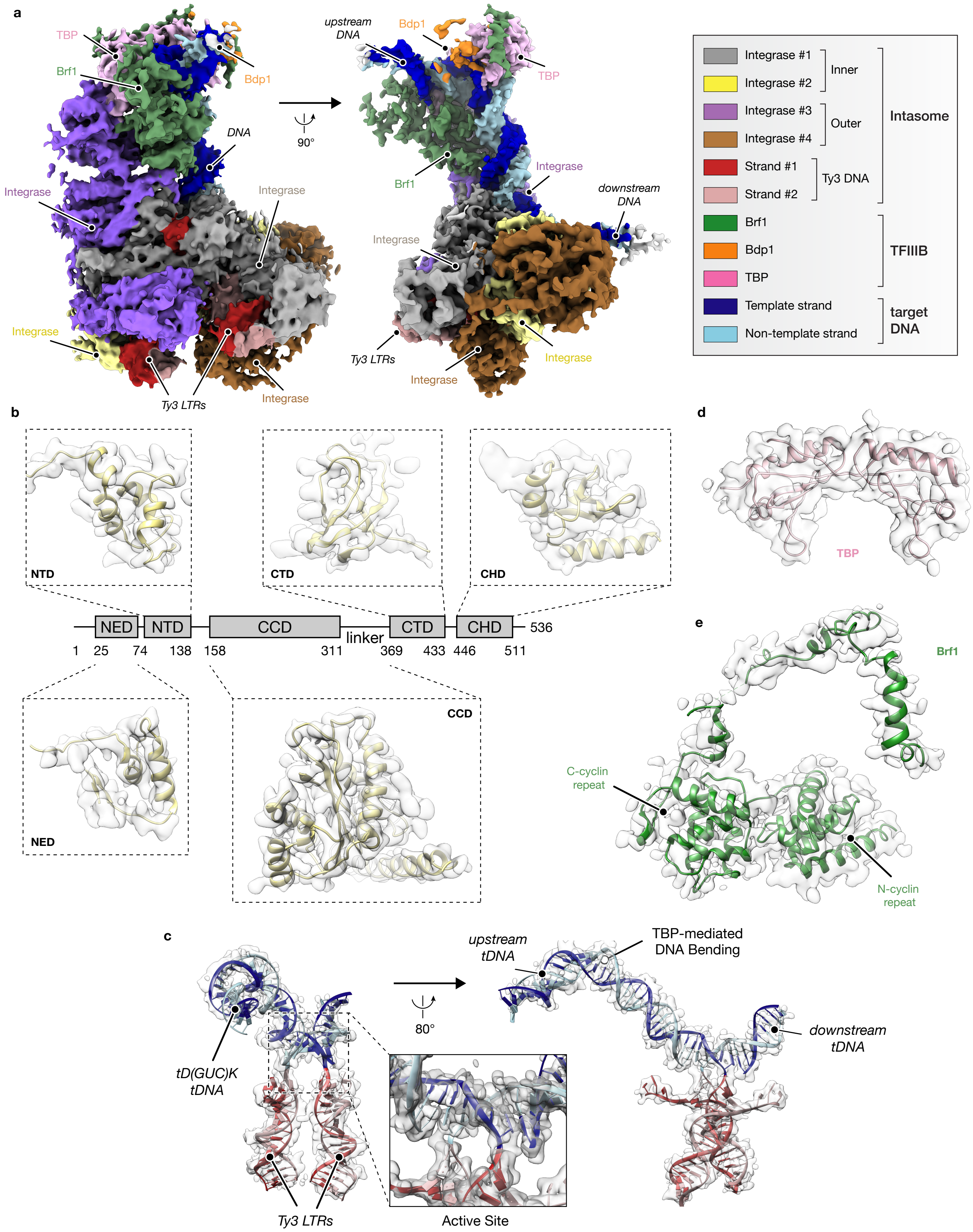

a, Cryo-EM reconstruction of the Ty3 retrotransposon targeting TFIIIB bound to tD(GUC)K DNA promoter. TFIIIB subunits, DNA molecules and Ty3 intasome monomers are coloured as indicated in the table. **b,** Domain architecture of Ty3 integrase. Cryo-EM maps are depicted in grey and protein models as yellow ribbons. **c,** Detail of DNA geometry and integration site. LTRs and target DNA are represented as ribbons in red and blue shades, respectively. **d,** TBP architecture. **e,** Brf1 domain architecture.

Supplementary Figure 4. Intasome organisation

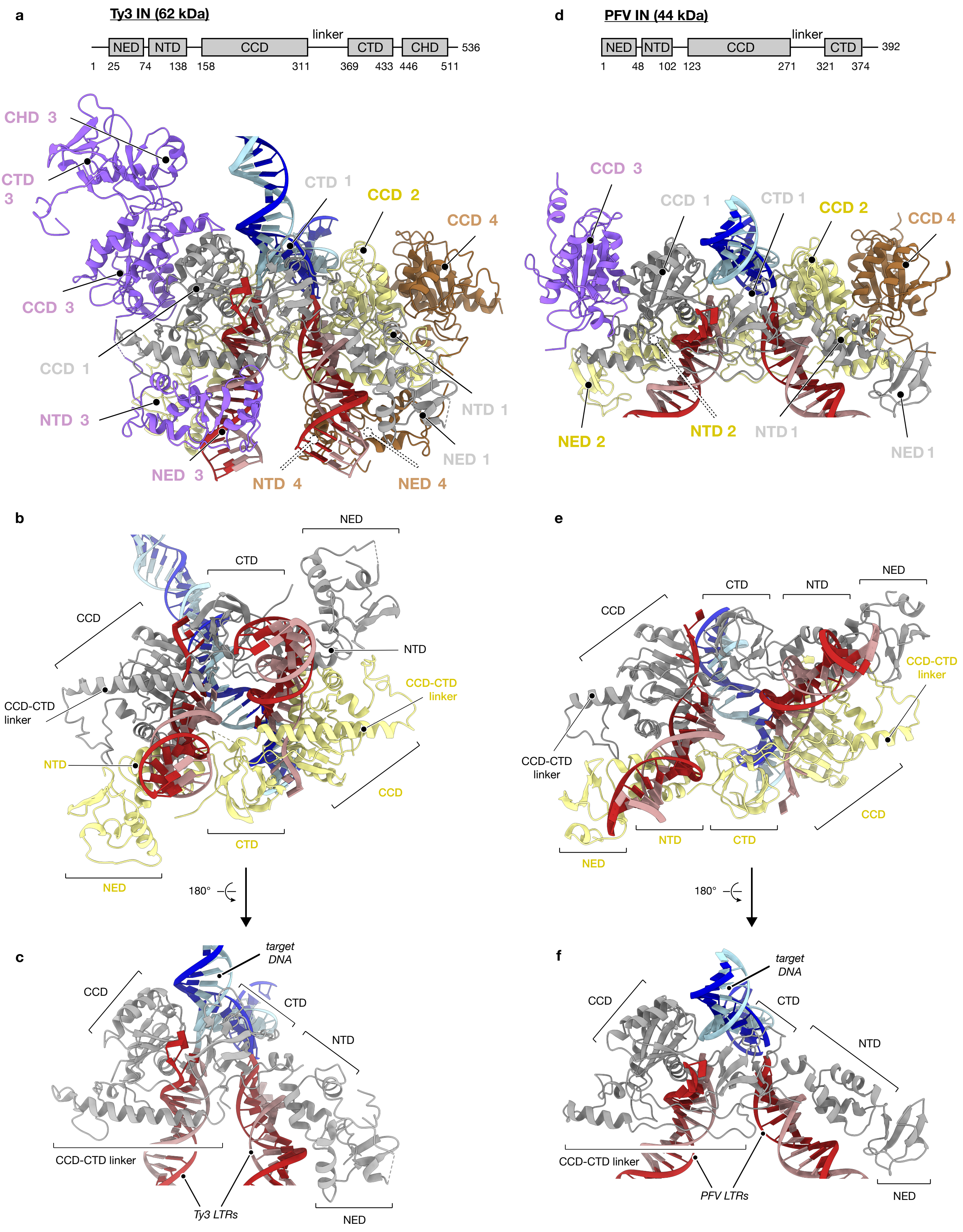

a, Schematic representation of Ty3 retrotransposon domain architecture (*inset*, molecular weight is indicated) and ribbon representation of Ty3 intasome structure model. Subunits are coloured and numbered as in Fig. 2: inner integrases 1 and 2 in grey and yellow, respectively; and outer integrases 3 and 4 in purple and brown, respectively. **b,** Ribbon representation of Ty3 retrotransposon inner subunits (yellow and grey) engaged with the target tD(GU-C)K DNA and the Ty3 gene LTR ends, forming the integration active site. Position of the integrase domains is indicated. **c,** Domain organisation detail of an isolated Ty3 integrase inner subunit (grey). **d-f,** As in **a-c,** but for Prototype foamy virus (PFV) integrase (pdb code: 3OS0).

Supplementary Figure 5. Chromodomain sequence and structure comparison

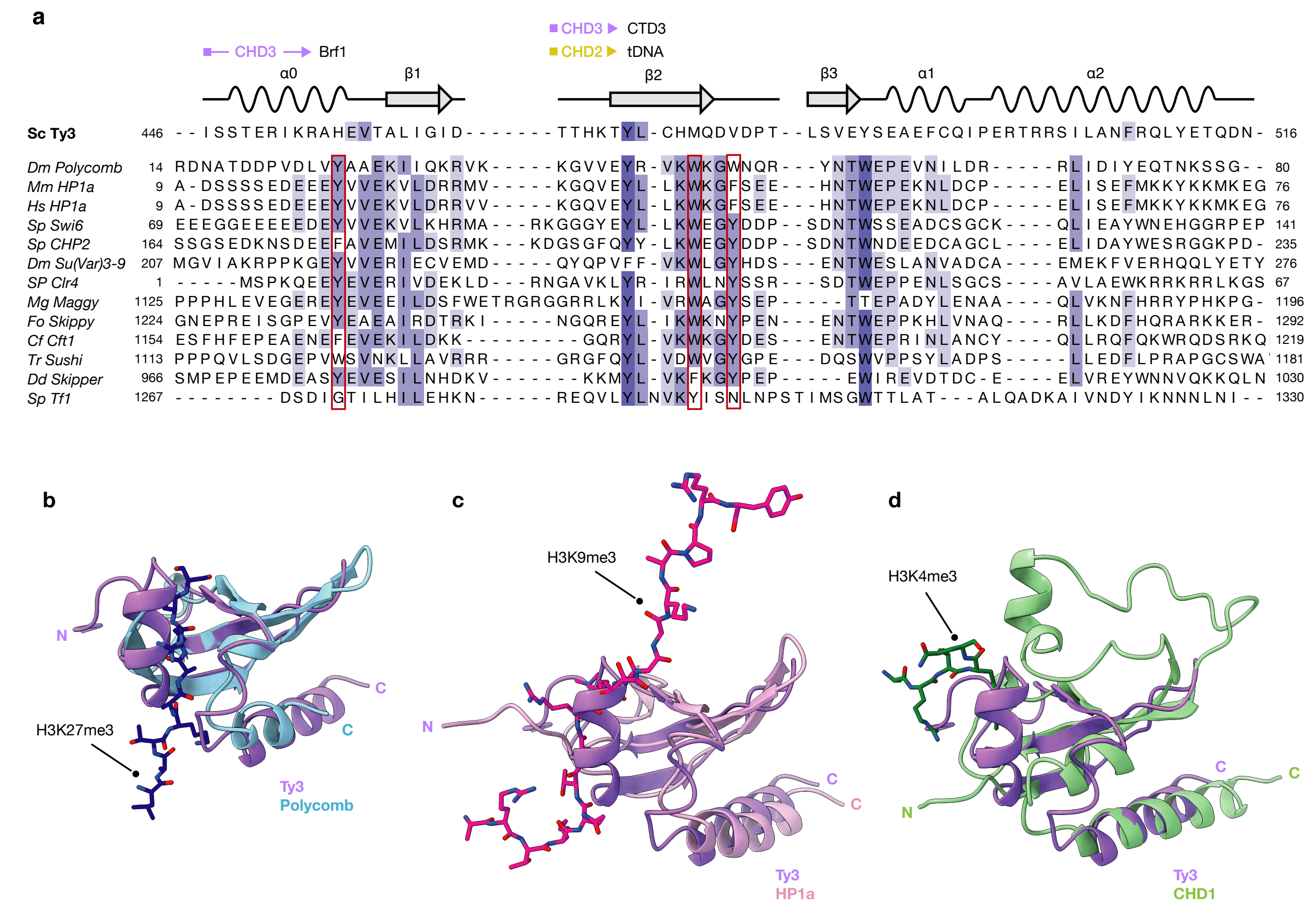

a, Sequence alignment of representative chromodomains from different species (Abbreviations: Sc, *Saccharomyces. cerevisiae*; Dm, *Drosophila melanogaster*; Hs, *Homo sapiens*; Sp, *Schizosaccharomyces pombe*; Mg, *Magnaporthe grisea*; Fo, *Fusarium oxysporum*; Cf, *Cladosporium fulvum*; Tr, *Takifugu rubripes*; Dd, *Dictyostelium discoideum*). The aromatic cage residues required for recognition of histone tail peptides are outlined with a red box. The schematic representation of Ty3 CHD motif organisation is shown above the sequence alignment. The regions of Ty3 chromodomains involved in interactions with other integrase domains or factors are delimited by an arrowed line, coloured and labelled according to the specific chromodomain subunit mediating the binding, as in Fig. 2c. Structure comparison of *S. cerevisiae* Ty3 integrase chromodomain (purple, this work) and **b,** *D. melanogaster* Polycomb chromodomain (light blue) complexed with H3K27me3 histone tail (dark blue) (PDB code: 1PDQ); **c,** *M. musculus* HP1a chromodomain (light pink) bound to the H3K9me3 histone tail (dark pink) (PDB code: 2RVN) and **d,** *H. sapiens* CHD1 first chromodomain (light green) complexed with H3K4me3 (dark green) (PDB code: 2B2W).

Supplementary Figure 6. Sequence comparisons

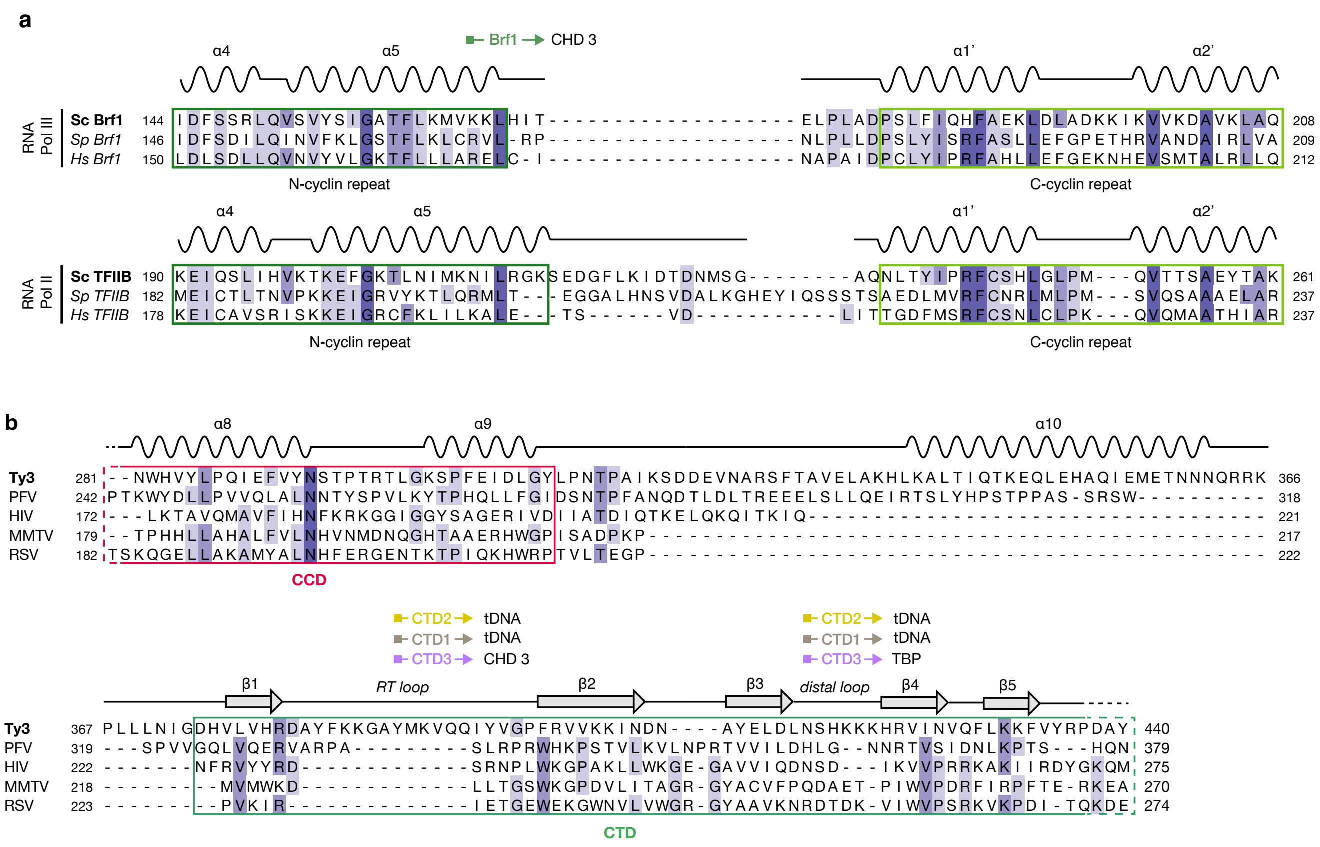

a, Multiple sequence alignments of the linker region between the B-core cyclin-repeats of Brf1 *(up)* and TFIIB *(down)* transcription factors in *S. cerevisiae*, *S. pombe* and *H. sapiens*. Secondary structure motifs are represented above each alignment. **b,** Multiple sequence alignment of the integrase CCD-CTD region of *S. cerevisiae* Ty3 and PFV, HIV, MMTV and RSV retroviruses. Secondary structure representation shows the position of the conserved motifs. Catalytic core domains (CCD) and C-terminal domains (CTD) are delimited by red and green boxes, respectively. The regions of Ty3 retrotransposon involved in interactions with other integrase domains or factors are delimited by an arrowed line, coloured and labelled according to the specific integrase subunit mediating the binding, as in Fig. 2c.

Supplementary Table 1

Cryo-EM data collection, refinement and validation statistics

**Data collection (0° Tilt)**	
Voltage (kV)	300
Electron exposure (e^{-}/Å^{2})	52.1
Defocus range (μm)	-1.7 to -3.2
Pixel size (Å)	1.047
**Data collection (30° Tilt)**	
Voltage (kV)	300
Electron exposure (e^{-}/Å^{2})	70.0
Defocus range (μm)	-2.4 to -4.0
Pixel size (Å)	1.06
**Reconstruction (RELION)**	
Initial particle images (no.)	628,998
Final particle images (no.)	101,469
Map resolution (Å)	3.98
FSC threshold	(0.143-thr)
Map sharpening *B* factor (Å^{2})	-157.407
**Model composition**	
Non-hydrogen atoms	21746
Protein residues	2255
Nucleotide residues	168
**Refinement (PHENIX)**	
Map CC (mask)	0.60
**R.m.s. deviations**	
Bond lengths (Å)	0.004
Bond angles (°)	0.875
**Validation**	
MolProbity score	2.11
Clashscore (all-atom)	9.05
Poor rotamers (%)	0.00
**Ramachandran plot**	
Favored (%)	86.85
Allowed (%)	13.15
Disallowed (%)	0.00
