# Supplementary material for: Structural basis of Ty3 retrotransposon integration at RNA Polymerase III-transcribed genes
Source: Nat Commun. 2021 Nov 30;12:6992. doi: 10.1038/s41467-021-27338-w (PMC8632968; doi:10.1038/s41467-021-27338-w)
Supplement: Supplementary file 3 — Reporting Summary [file 41467_2021_27338_MOESM3_ESM.pdf]

nature portfolio

Corresponding author(s): Abascal-Palacios, Guillermo
Vannini, Alessandro

Last updated by author(s): Nov 3, 2021



# Reporting Summary

Nature Portfolio wishes to improve the reproducibility of the work that we publish. This form provides structure for consistency and transparency in reporting. For further information on Nature Portfolio policies, see our Editorial Policies and the Editorial Policy Checklist.

## Statistics

For all statistical analyses, confirm that the following items are present in the figure legend, table legend, main text, or Methods section.

| n/a | Confirmed | |
|---|---|---|
| [ ] | [x] | The exact sample size (*n*) for each experimental group/condition, given as a discrete number and unit of measurement |
| [x] | [ ] | A statement on whether measurements were taken from distinct samples or whether the same sample was measured repeatedly |
| [x] | [ ] | The statistical test(s) used AND whether they are one- or two-sided<br>*Only common tests should be described solely by name; describe more complex techniques in the Methods section.* |
| [x] | [ ] | A description of all covariates tested |
| [x] | [ ] | A description of any assumptions or corrections, such as tests of normality and adjustment for multiple comparisons |
| [x] | [ ] | A full description of the statistical parameters including central tendency (e.g. means) or other basic estimates (e.g. regression coefficient) AND variation (e.g. standard deviation) or associated estimates of uncertainty (e.g. confidence intervals) |
| [x] | [ ] | For null hypothesis testing, the test statistic (e.g. *F*, *t*, *r*) with confidence intervals, effect sizes, degrees of freedom and *P* value noted<br>*Give P values as exact values whenever suitable.* |
| [x] | [ ] | For Bayesian analysis, information on the choice of priors and Markov chain Monte Carlo settings |
| [x] | [ ] | For hierarchical and complex designs, identification of the appropriate level for tests and full reporting of outcomes |
| [x] | [ ] | Estimates of effect sizes (e.g. Cohen's *d*, Pearson's *r*), indicating how they were calculated |

*Our web collection on statistics for biologists contains articles on many of the points above.*

## Software and code

Policy information about availability of computer code

| | |
|---|---|
| Data collection | EPU 2 Software (Thermo Fisher Scientific) |
| Data analysis | Relion (versions 3.0.4 to 3.1.1); MotionCor2; CFFIND4; CryoSPARC 2.0; PsiPred webserver; Phenix Suite (version 1.18.1-3865); Coot (version 0.8.9.2); UCSF Chimera 1.14 and ChimeraX-1.0; Clustalw Omega webserver; Jalview 2.10.1; Pymol v1.8.6.0 |

For manuscripts utilizing custom algorithms or software that are central to the research but not yet described in published literature, software must be made available to editors and reviewers. We strongly encourage code deposition in a community repository (e.g. GitHub). See the Nature Portfolio guidelines for submitting code & software for further information.

## Data

Policy information about availability of data

All manuscripts must include a data availability statement. This statement should provide the following information, where applicable:

- Accession codes, unique identifiers, or web links for publicly available datasets
- A description of any restrictions on data availability
- For clinical datasets or third party data, please ensure that the statement adheres to our policy

The structure of the Ty3 intasome engaged with TFIIIB and the tRNA promoter and its associated data have been deposited into the Protein Data Bank under accession code 7Q5B, and into the Electron Microscopy Data Bank under accession code EMD-13831. Additional atomic models used in this study correspond to: PFV strand transfer complex (3OS0), HIV-1 Strand Transfer Complex (5U1C), HIV-1 integrase and LEDGF/p75 (2B4J), RSV intasome (5EJK), Mouse Mammary Tumour Virus intasome (3JCA), HTLV-1 intasome (6VOY), RNA Polymerase III open pre-initiation complex (6EU0), NC2 in complex with TBP and Mot1 N-terminal domain (4WZS), Polycomb chromodomain complexed with H3K27me3 histone tail (1PDQ), HP1a chromodomain bound to the H3K9me3 histone tail (2RVN) and CHD1 first chromodomain complexed with H3K4me3 (2B2W). Source data are provided as a Source Data file with this paper.

# Field-specific reporting

Please select the one below that is the best fit for your research. If you are not sure, read the appropriate sections before making your selection.

☒ Life sciences ☐ Behavioural & social sciences ☐ Ecological, evolutionary & environmental sciences

For a reference copy of the document with all sections, see nature.com/documents/nr-reporting-summary-flat.pdf

# Life sciences study design

All studies must disclose on these points even when the disclosure is negative.

| | |
|---|---|
| Sample size | No statistical methods were used to predetermine sample size. The total amount of data collected during the experiment was the total data available for analysis in each case. |
| Data exclusions | No data were excluded initially from the analysis. During cryoEM analysis, picked coordinates which did not correspond to protein particles were discarded after 2D classification. Particles which did not correspond to the complete Ty3 targeting complex were discarded following global and local 3D classification. |
| Replication | Experimental findings were reproduced as 4 independent cryo-EM data sets, 4 of which were then merged to give the final maximally resolved structure. The resulting structure is an average of 101469 individual complex particles following 2D and 3D classification. |
| Randomization | N/A to this study as purifications were directed to isolate specific proteins of interest and in structural determination all data is included in a single analysis. |
| Blinding | N/A for cryoEM analysis, as purified samples were prepared to produce standard grids or crystals which were compatible for structural determination using these techniques. Subsequent data collection and analysis is then automated and does not require blinding. |

# Reporting for specific materials, systems and methods

We require information from authors about some types of materials, experimental systems and methods used in many studies. Here, indicate whether each material, system or method listed is relevant to your study. If you are not sure if a list item applies to your research, read the appropriate section before selecting a response.

## Materials & experimental systems

| n/a | Involved in the study | |
|---|---|---|
| ☒ | ☐ | Antibodies |
| ☐ | ☒ | Eukaryotic cell lines |
| ☒ | ☐ | Palaeontology and archaeology |
| ☒ | ☐ | Animals and other organisms |
| ☒ | ☐ | Human research participants |
| ☒ | ☐ | Clinical data |
| ☒ | ☐ | Dual use research of concern |

## Methods

| n/a | Involved in the study | |
|---|---|---|
| ☒ | ☐ | ChIP-seq |
| ☒ | ☐ | Flow cytometry |
| ☒ | ☐ | MRI-based neuroimaging |

## Eukaryotic cell lines

Policy information about cell lines

| | |
|---|---|
| Cell line source(s) | 1. Sf9 insect cells<br>2. Hi5 insect cells |
| Authentication | Sf9 and Hi5 insect cells were not authenticated as part of this study. |
| Mycoplasma contamination | The cell lines were not tested for mycoplasma contamination. |
| Commonly misidentified lines (See ICLAC register) | None |
